# Immunologic Response of HIV-Infected Children to Different Regimens of Antiretroviral Therapy: A Retrospective Observational Study

**DOI:** 10.1155/2020/6415432

**Published:** 2020-08-13

**Authors:** Teshale Ayele Mega, Firehiwot Belayneh Usamo, Getandale Zeleke Negera

**Affiliations:** ^1^Department of Clinical Pharmacy, School of Pharmacy, Institute of Health, Jimma University, Jimma, Ethiopia; ^2^Department of Pharmacy, College of Medicine and Health Science, Dilla University, Dila, Ethiopia

## Abstract

**Background:**

Both abacavir- (ABC-) based and zidovudine- (AZT-) based regimens are widely utilized for managing HIV infection in children. Unfortunately, there is a lack of data regarding their immunological response and associated risk factors in Ethiopia.

**Methods:**

A retrospective hospital-based cohort study was conducted on HIV-infected children in Jimma Medical Center (JMC). A total of 179 records were reviewed by including data from November 2015 to April 2017. Data were collected on sociodemographic, clinical characteristics of patients and drug-related variables. Data analysis was done using STATA 13.1. Mixed-effect linear regression was performed to assess the difference in CD4+ changes between groups adjusting for baseline characteristics. The change in predicted CD4 count attributed to each regimen was also assessed by marginal analysis. *P* < 0.05 for slope of the random-effect linear regression was used as an indicator for the presence of association.

**Result:**

Of 179 patients, 98 (54.7%) were females. The mean (±SD) duration of follow-up was 939.8 ± 478.3 and 984.92 ± 453.1 days for ABC and AZT groups, respectively. AZT group had a significant CD4+ count gain per visit compared with their ABC counterparts ((*β* = 20.51, 95% CI [6.37–34.65]), *P* = 0.004) over time. The regimen AZT + 3TC + LPV/r tended to have an excellent predicted CD4+ lymphocyte count change relative to all other regimens, while ABC + 3TC + LPV/r had the least immunologic recovery (margins 338.0 cells/mm^3^ versus 249.13 cells/mm^3^ (*P* < 0.001)). Baseline CD4+ lymphocyte count, ART group, WHO clinical stages, and viral load were independent predictors for CD4+ change overtime.

**Conclusion:**

AZT-based regimens seem to have better immunological response compared to ABC-based regimens. Immunologic response was described worse in patients with a viral load of >1000copies/ml, low baseline CD4+ count, advanced WHO clinical stages, and ABC-containing regimens. Further study is needed to clarify these aspects.

## 1. Background

Currently, 1.7 million children are living with human immunodeficiency virus (HIV) and over 90% of them live in sub-Saharan Africa [1, 2]. Ethiopia has the largest population of HIV-infected children in the region. According to an estimate by the Federal HIV/AIDS Prevention and Control Office (FHAPCO), there are over 738,976 people living with HIV/AIDS in Ethiopia [3]. Of these, 178,500 are children younger than 15 years of age [4].

The initiation of potent antiretroviral therapy (ART) in the mid-1990s has dramatically reduced HIV-associated morbidity and mortality [5–8]. Antiretroviral therapy reduces HIV-associated morbidity and mortality by suppressing HIV replication to undetectable levels and providing a consistent increase in the number of CD4+ T lymphocytes [9, 10]. The resulting effect is the recovery of the immune system, and thus, immune reconstitution is an important outcome measure of ART [11]. According to the recent report, 59% of children living with HIV were on treatment in Ethiopia [12].

The earlier World Health Organization (WHO) guidelines recommended using a nucleoside reverse transcriptase inhibitor (NRTI) backbone including a thymidine analogue stavudine (d4T) or zidovudine (AZT) together with lamivudine (3TC) for paediatric HIV treatment [13]. But in 2010, the WHO guideline discouraged the use of d4T because of high lipodystrophy rates in adults and adolescents [14]. As a result, the current guidelines were changed to recommend substitution of the thymidine analogue with abacavir (ABC) depending on studies which reported fewer side effects and improved virological responses with ABC compared to d4T or AZT [15, 16].

In Ethiopia, the initial ART regimen included the use of d4T as a preferred first-line NRTI for paediatric HIV treatment. In 2012, Ethiopia implemented the d4T phase-out program, and ABC became routinely used as a standard of care [17]. Currently, both ABC- and AZT-based regimens are routinely utilized. However, there is a lack of study regarding the immunological response of ABC- and AZT-containing regimens among HIV-infected children in Ethiopia. Studies conducted in some African children [18–21] showed an encouraging and comparable immunologic response to those obtained among children in well-resourced countries [11, 22, 23]. In this study, we compared the immunological response and associated factors in a cohort of HIV-infected children receiving ABC- and AZT-containing regimens.

## 2. Methods

### 2.1. Study Design and Setting

A retrospective hospital-based cohort study was conducted on HIV-infected children in Jimma Medical Center (JMC). JMC is located in Jimma town, 355 km from Addis Ababa. It is currently the only teaching and specialized hospital in the Southwest region of Ethiopia. The hospital serves as a referral site and provides specialized care for Southwest Ethiopia with a catchment population of about 15 million. The study was conducted from April 10 to May 10, 2017, by including data from November 2015 to April 2017.

### 2.2. Study Population and Variables

We included HIV-infected children (<15 years) who were on ABC- and AZT-based regimens that fulfill the eligibility criteria. Patients on ABC- and AZT-based first-line regimens, having at least six months of follow-up with good adherence, whose records were legible and complete, who have CD4 count at least at the baseline and six months, and younger than 15 years were included in the study. Those transferred out within <6 months of follow-up and patients with incomplete records were excluded. The study was conducted by dividing the total sample into two major classes as ABC group and AZT group.

Data were collected on sociodemographic characteristics (age, sex, area of residence, weight (kg), height (cm), and body mass index (BMI)), HIV-related factors (CD4 count and WHO clinical stage), treatment-related factors (types of ART regimen, opportunistic infection (OI) prophylaxis (cotrimoxazole preventive therapy (CPT) and isoniazid preventive therapy (IPT)), and antitubercular treatment), and immunological response (CD4+change).

### 2.3. Sample Size Determination

The number of patients who fulfilled the eligibility criteria for the ABC group was 87 and all of them were included. From those on AZT-based regimen, 92 eligible patients were included, making ABC-to-AZT group ratio of 1 : 1.05. Finally, a total of 179 subjects, with 87 charts of patients from the ABC group and 92 patients from the AZT group were reviewed.

### 2.4. Sampling Technique

Since the number of patients on ABC-based regimen was limited, we included all eligible patients. A simple random sampling technique was used to select 92 patient charts for AZT-based regimens using a computer-generated random number.

### 2.5. Data Collection Procedure and Quality Assurance

Data on socio-demographic, clinical, laboratory, and drugs administered were collected by record review using English version checklists. The data collection tool was carefully prepared after reviewing relevant literature studies to enable the data collectors to gather all the information required to address the study objectives. A 2-day training was provided on the data collection tool and general procedures for data collectors, i.e., 2 pharmacists (B. Pharm) and 2 nurses. Data from antiretroviral drugs and patient information sheet were collected by pharmacists, and data from ART clinic intake form, HIV care/ART follow-up, and patient sheet were collected by the nurses. The baseline body mass index of the subjects was later calculated after collection of baseline height and weight of the patient from the patient chart. Pretest was conducted on 5% of the eligible records.

### 2.6. Operational Definition of Terms

  Good adherence: estimated adherence level of >95% [24] as recorded by ART physicians/nurses
  Child: age <15 years [25]
  Lost to follow-up: refer to a patient who has missed clinical or drug pickup appointments permanently [26].

### 2.7. Ethical Consideration

The study was approved by the Institutional Review Board (IRB) of Jimma University. It has designated with an IRB number of IHRPGB/112/2017. The need for informed consent was waived because of the retrospective, anonymous nature of the study. During data collection, confidentiality was ensured, and for this reason, the name and address of the patient were not recorded in the data collection checklist.

### 2.8. Statistical Analysis

Data were double-entered into Epi-Data and exported to STATA 13.1 for cleaning and analysis. Descriptive analysis was performed, and results were presented in text, tables, and charts. Bivariate and multivariate mixed-effects linear regressions for repeated measurements were performed to assess the adjusted effect of the ART regimen and identify additional predictors of CD4+ recovery. The coefficient of mean CD4+ count with 95% confidence intervals was used as a measure of the strength of association, and *P* < 0.05 was considered to declare a statistical significance. Marginal analysis was also conducted to see the difference among the specific regimen category.

## 3. Results

### 3.1. Overview of the Study Participants

**Figure 1:**
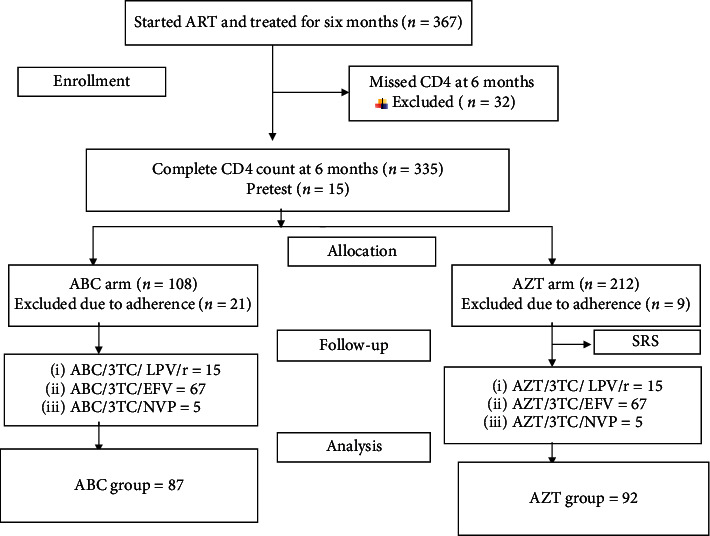
Sample recruitment chart of patients attending ART clinic, in JMC, from April 10 to May 10, 2017. SRS: simple random sampling.

During the study period, a total of 367 patients started antiretroviral therapy (ART) and were treated for at least 6 months. Of these, 108 from ABC group and 212 from AZT group have a complete CD4+ count at the 6^th^month of treatment. Thirty-two patients were excluded initially from either regimen due to missed CD4+ count at 6 months and 30 (21 and 9 from ABC and AZT, respectively) because of the adherence problem, and 179 patients were included in the analysis (Figure 1).

The mean ± standard deviation (SD) duration of follow-up was 939.8 + 478.3 and 984.92 + 453.1 days for the ABC and AZT groups, respectively. During the study period, a total of 4 patients (1 (1.14%) patient from the ABC group and 3 (3.3%) from the AZT group; *P*=0.339) died.

### 3.2. Baseline Socio-Demographic and Clinical Characteristics

We included 179 patients, of whom 98 (54.75) were females. One hundred forty (78.2%) of the patients had a BMI of less than fifth centile, and there was a statistically significant difference between groups (*P* = 0.03). Baseline socio-demographic and clinical characteristics of the study participants are depicted in Table 1.

### 3.3. Immunological Response

**Figure 2:**
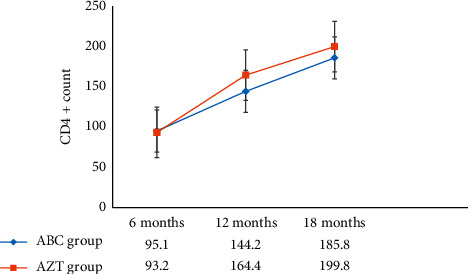
Comparative mean CD4+ count gains for paediatric patients exposed to ABC versus AZT from April 10 to May 10, 2017.

The overall change in the mean standard deviation (±SD) of CD4+ lymphocyte count was 139.3 (±55.7) cells/mm^3^ for the ABC group and 150.5 (±42.1) cells/mm^3^ for the AZT group. The mean change in CD4+ lymphocyte count from baseline for the past 18 months was presented comparatively in Figure 2. The SDs are 55.3 vs. 41.6, 66.3 vs. 63.1, and 87.7 vs. 65.1 at 6^th^, 12^th^, and 18^th^ months for ABC- and AZT-based regimens, respectively.

As shown in the figure, the CD4+ lymphocyte count trajectory belonging to AZT-based regimen looks steeper after the first 6 months of therapy.

Patients on AZT + 3TC + NVP had the highest baseline CD4+ lymphocyte count, followed by AZT + 3TC + LPV/r and ABC + 3TC + NVP. The lowest baseline CD4+ count was recorded for ABC + 3TC + LPV/r (Table 2).

### 3.4. Predictors of CD4+ Lymphocyte Count Change

We conducted a mixed-effect linear regression using a consecutive CD4+ lymphocyte count measured over the past 18 months as an outcome variable. The slope of random-effect linear regression was used to report the overtime change in CD4+ count attributed to the predictor variables. The overall average gain in CD4+ count every six months was 53.0 cells/mm^3^ (*P* < 0.001 and 95% CI [46.86–59.20]) with an interclass correlation coefficient of 65.5% (*P* < 0.001). This implies a significant proportion of variability in CD4+ lymphocyte count change due to patient-specific factors. Among the groups, 36.73% of the variation in CD4+ lymphocyte change variability was explained by differences in ART regimens (*P* < 0.001).

Body mass index, weight for height, ART group, baseline, CD4+ lymphocyte count, occurrence of opportunistic infections (OIs), WHO clinical staging, viral load, nutritional status, and exposure to antitubercular drugs were associated with CD4+ lymphocyte count change on binary linear regression. After adjusting for all confounders, ART group, baseline CD4+ lymphocyte count, WHO clinical stages, and viral load remained independent predictors for CD4+ lymphocyte count change.

Therefore, patients who commenced on AZT-based regimens had a significant change in CD4+ lymphocyte count at each visit. Accordingly, patients exposed to AZT-based regimens had 20.51 cells/mm^3^ CD4+ count advantage every six months (*β* = 20.51, 95% CI [6.37–34.65]). Each unit increment in baseline CD4+ count will contribute to 0.55 cells/mm^3^ CD4 gain every half year (*β* = 0.55, 95% CI [0.49–0.69]).

On the contrary, patients with WHO stages III and IV were in a precarious situation in terms of their CD4+ count recovery. Every six months, patients with WHO stage III and WHO stage IV had 47.61 and 73.54 CD4+ lymphocyte count disadvantages, respectively (*β* = −47.61[−84.27− (−10.96)] and *β* = −73.54[−118.27− (−28.81)]). In addition, patients with a viral load of >1000 copies/ml had 28 CD4+ lymphocyte count disadvantages every half year (*β* = −27.68[−47.75− (−7.61)]) (Table 3).

### 3.5. Postestimation Treatment Effects

We also conducted a marginal analysis to predict the changes in the mean CD4+ lymphocyte counts associated with each regime and to identify the regimen with the lowest performance. Therefore, except ABC + 3TC + NVP, the other two ABC-based regimens, namely, ABC + 3TC + EFV and ABC + 3TC + LPV/r, deemed to be the lowest performing regimens as compared to their AZT counterparts. Accordingly, the predicted mean CD4+ count for paediatrics treated with AZT + 3TC + NVP had 318.3 cells/mm^3^ of CD4+ lymphocyte count change, and it was 296.33 cells/mm^3^ CD4+ for those treated with ABC + 3TC + EFV. The change is statistically significant in either case (*P* < 0.001). The regimen AZT + 3TC + LPV/r tended to have an excellent predicted CD4+ lymphocyte count change relative to all other regimens, while ABC + 3TC + LPV/r performed the opposite (margins 338.0 cells/mm^3^ versus 249.13 cells/mm^3^ (*P* < 0.001)) (Table 4).

## 4. Discussion

This study was the first ever to compare the immunological response of ABC- and AZT-based regimens among HIV-infected children in Ethiopia. The biannual analysis of mean CD4+ gain showed that the maximum gain in mean CD4+ count was attained with AZT + 3TC + LPV/r, while ABC + 3TC + LPV/r had the least immunologic recovery over the entire treatment course. The CD4+ lymphocyte count trajectory showed a linear trend. Baseline CD4+ lymphocyte count, ART group, WHO clinical stages, and viral load were independent predictors for CD4+ changes over time.

Our finding of mean CD4+ recovery was inconsistent with a randomized open-label study by Mulenga et al. [27], where a comparable immunologic response was achieved between AZT- and ABC-containing regimens. A study by Cassim et al. [28] also failed to show a significant difference in terms of immunologic response between ART regimens. The higher CD4+ recovery with AZT-based regimens in this study might be due to a higher baseline CD4+ count of those patients who were on AZT-containing regimens (Table 1). In addition, the difference in the study design might contribute to the variation. This is thus an additional evidence to advocate to the test and treat strategy as opposed to waiting for dropping of CD4 counts at lower thresholds [24]. The finding from the marginal analysis also indicated that the change in mean CD4+ count is significantly higher in AZT-based regimens. Adult immunologic studies indicated that a linear trend in CD4+ increment at the early phase of therapy is expected and it became flat with minimal CD4+ gain later after 18^th^ month of treatment course [29]. However, the linear trend of immunologic recovery may indicate our study was ended prematurely.

The overall random-effect linear regression analysis had pointed out that baseline CD4+ lymphocyte count, ART group, WHO clinical stages, and viral load were independent predictors for CD4+ change overtime.

The marginal effects of each regimen confirmed that the immunologic outcome associated with AZT+3TC + LPV/r (margins = 338.0 cells/mm3/, *P* < 0.001) was the most favorable followed by ABC+3TC + NVP (*m* = 337.8 cells/mm3, *P* < 0.001) and AZT + 3TC + EFV (*m* = 320.3, *P* < 0.001). However, ABC+3TC + LPV/r had the lowest predicted change in CD4+ count (*m* = 249.13, *P* < 0.001), implying minimal immunologic response, and further study is needed to clarify this finding.

Patients who commenced on AZT-based regimens had greater CD4+ improvement over time. Accordingly, those patients who had exposed to AZT-based regimens were associated with an average CD4+ count advantage of 20.51 cells/mm^3^ (*β* = 20.51, 95% CI [6.37–34.65]). This finding is inconsistent with the Paediatric European Network for the Treatment of AIDS (PENTA 5) study, where children on ABC-based regimens had better immunologic response as compared to AZT-containing regimens [30]. This variation might be due to differences in study design (RCT vs. retrospective), study setup, and sample size.

In the cohort, baseline CD4+ count was another positive predictor for successful immunologic recovery. Therefore, each unit increment in the baseline CD4+ count will contribute to 0.55 cells/mm^3^ CD4 gain every half year (*β* = 0.55, 95% CI [0.49–0.69]). This is concurrent with previous studies [31–34], in which a higher baseline CD4+ lymphocyte count was associated with better immunologic response. This could be due to less extensively depleted immune system, which will be boosted easily after initiation of ART. It is also in agreement with the current WHO ART guidelines [35], which recommends the initiation of ART at diagnosis (test and treat) regardless of CD4+ count and WHO stage.

On the contrary, patients with WHO stages III and IV were in a precarious situation in terms of their CD4+ count recovery. Every six months, patients with WHO stage III and WHO stage IV had 47.61 and 73.54 CD4+ lymphocyte count disadvantage, respectively (*β* = −47.61[−84.27− (−10.96)] and *β* = −73.54[−118.27− (−28.81)]). This is in agreement with studies conducted in Nigeria [36] and Tanzania [37], in which advanced clinical stage was associated with worse CD4+ recovery. Similar observation was also reported by Kaufmann et al. [38], in which patients with advanced HIV stage had poor immune recovery compared to those in the early stage of the disease. This finding suggests that patients with advanced HIV/AIDS and profound immune suppression need special attention to improve their outcome. Similarly, patients with a viral load of >1000 copies/ml had a worse immunologic response (*β* = −27.68[−47.75− (−7.61)]). This finding is consistent with the study conducted by Zhou et al. [39].

This study had several possible limitations. Firstly, because of the retrospective nature of the study, we were not able to capture some important data such as adverse drug effects of ART. Second, as the sample size was relatively small, the power to detect definitive differences may have been limited. It was a single-site hospital-based study, and therefore, the findings may not be generalizable to the general population, and measures of adherence by health professionals that may not fit the reality are some of the limitations.

## 5. Conclusion

In the current study, patients with AZT + 3TC + LPV/r had a better immunologic recovery. Immunologic response was described worse in patients with a viral load of >1000 copies/ml, low baseline CD4+ count, advanced WHO clinical stages, and ABC-containing regimens. Further study is needed to clarify these aspects.

## Figures and Tables

**Table 1:** Comparative baseline characteristics of the cohort at JMC, from April 10 to May 10, 2017.

All *n* = 179		ABC group (*n* = 87)	AZT group (*n* = 92)	*P* value
Variables				
Sex	Male	42 (48.3%)	39 (42.4%)	0.42
	Female	45 (51.7%)	53 (57.6%)	

Age (years)	<3 years	11 (12.6%)	12 (13.0%)	0.97
	3–5 years	18 (20.7%)	20 (21.7%)	
	>5 years	58 (66.7%)	60 (65.2%)	

BMI (baseline)	<5^th^ centile	74 (85.0%)	66 (71.7%)	0.03
	>5^th^ centile	13 (14.9%)	26 (28.3%)	

Maternal HIV status	Positive	78 (89.7%)	83 (90.2%)	0.91
	Unknown	9 (10.3%)	9 (9.8%)	

Area of residence	Urban	66 (75.9%)	68 (73.9%)	0.76
	Rural	21 (24.1%)	24 (26.1%)	

Baseline CD4+ (mean +SD)	166.31 + 76.223	178.78 + 71.12	0.26	

WHO stage	I	8 (9.2%)	3 (3.3%)	0.08
	II	24 (27.6%)	40 (43.5%)	
	III	45 (51.7%)	42 (45.7%)	
	IV	10 (11.5%)	7 (7.6%)	

Functional status	W/A	72 (82.8%)	88 (95.7%)	0.001
	A/D	12 (13.8%)	0	
	B/r	3 (3.4%)	4 (4.3%)	

TB (treatment)	Yes	3 (3.4%)	9 (9.8%)	0.06
	No	84 (96.6%)	83 (90.2%)	

OI prophylaxis	Both CPT and INH	85 (97.7%)	89 (96.7%)	0.69
	CPT only	1 (1.1%)	1 (1.1%)	
	Neither	1 (1.1%)	2 (2.2%)	

Nutritional status	Normal	45 (51.7%)	57 (62.0%)	0.17
	SAM	42 (48.3%)	35 (38.0%)	

**Table 2:** CD4+ lymphocyte count at different points of time with respect to each regimen.

Regimen	At baseline	6 months	12 months	18 months
ABC + 3TC + LPV/r, mean (±SD)	133.0 (8.01)	94.4 (14.18)	165.7 (21.9	162.83 (56.4)
ABC + 3TC + EFV	171.2 (5.59)	95.2 (6.80)	142.59 (9.9)	195.10 (18.4)
AZT + 3TC + NVP	221.0 (9.16)	68.6 (20.86)	116.3 (22.6)	135.3 (17.6)
AZT + 3TC + LPV/r	191.67 (10.83)	91.2 (10.2)	161 (14.4)	217.6 (11.0)
AZT + 3TC + EFV	174.2 (4.88)	95.1 (4.98)	165.4 (8.4)	197.3 (10.2)
ABC + 3TC + NVP	186.8 (11.95)	99 (22.58)	145.8 (25.1)	208.2 (31.1)

**Table 3:** Random-effect linear regression analysis of trend of CD4+ count (slope, cells/mm^3^/6 month) at JMC, from April 10 to May 10, 2017.

Variables		*n* (%)	Unadjusted %*β* [95%CI]	*P* value	Adjusted %*β* [95%CI]	*P* value
Sex	Male	98 (54.75)	0			
	Female	81 (45.25)	7.46[−13.61−28.53]	0.488		

Age: median (IQR)	7 (4–9)	179 (100)	−0.56[−4.24−3.12]	0.765		

BMI	Below 5^th^ centile	140 (78.21)	−19.45[−44.56−5.70]	0.130	−5.18[−22.99−12.63]	0.569
	Above 5^th^ centile	39 (21.79)	0		0	

Weight for height	≤70%	36 (20.11)	−31.84[−57.96− (−5.72)]	0.017	2.31[−18.10−22.72]	0.825
	70–85%	14 (7.82)	−9.84[−48.62−28.94]	0.619	5.91[−21.17−32.99]	0.669
	≥85%	129 (72.07)	0		0	

In care of the child	Mother	24 (13.41)	16.91[−13.74−47.57]	0.280		
	Others	155 (86.59)	0			

Maternal status	Dead	33 (18.44)	−3.244[−30.53−24.06]	0.816		
	Live	146 (8156)	0			

Maternal serostatus	Unknown	18 (10.06)	−5.01[−40.54−30.53]	0.782		
	Negative	161 (89.94)	0			

Paternal status	Dead	47 (26.26)	0.32[−23.39−24.03]	0.979		
	Live	132 (73.74)	0			

Residence	Urban	134 (78.86)	0			
	Rural	45 (25.14)	−6.59[−31.04−17.87]	0.598		

ART group	ABC	87 (48.60)	0		0	
	AZT	92 (51.40)	30.42[9.89–50.94]	0.004	20.51[6.37−34.65]	0.004

CD4 count	162 (117–221)	179 (100)	0.72[0.62–0.82]	≤0.001	0.55[0.49−0.69]	≤0.001
OI occurred	No	121	0		0	
	Yes	58	−49.12[−70.49− (−27.74)]	≤0.001	−18.65[−5.49−42.78]	0.130

Viral load	≤1000 copies/ml	146 (83.43)	0			
	>1000 copies/ml	29 (16.57)	−50.21[−77.71− (−22.71)]	≤0.001	−27.68[−47.75− (−7.61)]	0.007

WHO stage	Stage I	11 (6.15)	0		0	
	Stage II	64 (35.75)	−31.43[−73.23−10.37]	0.141	−28.63[−64.05−6.71]	0.112
	Stage III	87 (48.60)	−94.64[−136.08− (−53.19)]	≤0.001	−47.61[−84.27− (−10.96)]	0.011
	Stage IV	17 (9.50)	−151.41[−199.26− (−103.56)]	≤0.001	−73.54[−118.27− (−28.81)]	0.001

Nutritional status	Normal	102 (57.00)	0		0	
	SAM	77 (43.00)	−38.79[−59.28− (−18.30)]	≤0.001	−13.42[−35.34−8.50]	0.230

TB treatment	No	166 (92.74)	0		0	
	Yes	13 (7.26)	−61.14[−103.84− (−18.43)]	0.005	−32.58[−65.18−0.21]	0.050

**Table 4:** The predicted CD4+ lymphocyte count change of paediatric patients receiving ABC- and AZT-based regimens at JMC.

ART regimens	Delta method		*t*	*P* value	95% CI
	Margins	Standard error			
AZT + 3TC + NVP	318.3	25.38	12.54	*P* < 0.001	268.38–368.17
AZT + 3TC + EFV	320.3	6.40	50.05	*P* < 0.001	307.73–332.89
AZT + 3TC + LPV/r	338.0	14.23	23.76	*P* < 0.001	310.03–365.97
ABC + 3TC + NVP	337.8	21.73	15.54	*P* < 0.001	295.07 380.53
ABC + 3TC + EFV	296.33	7.22	41.05	*P* < 0.001	282.14–310.52
ABC + 3TC + LPV/r	249.13	15.12	16.48	*P* < 0.001	219.41–278.85

## Data Availability

The data used to support the findings of this study are available from the corresponding author on request.

## Abbreviations

ART: Antiretroviral therapy
CD4+: Cluster of differentiation 4
CI: Confidence interval
CPT: Cotrimoxazole preventive therapy
HIV: Human immunodeficiency virus
JMC: Jimma Medical Center
SD: Standard deviation.
